# Spatial and temporal differences of Chinese tourists’ travel demands to North Korea

**DOI:** 10.1371/journal.pone.0272731

**Published:** 2022-10-03

**Authors:** Yuanyuan Li, Guangyi Jin, Boyang Sun, Zhehao Cui, Bishun Lu

**Affiliations:** 1 Yanbian University, College of Economics and Management, Hunchun, Peoples Republic of China; 2 Yanbian University, College of Geography and Ocean Science, Hunchun, Peoples Republic of China; 3 Yanbian University, College of Integration Science, Yanji, Peoples Republic of China; Chinese Academy of Sciences, CHINA

## Abstract

Border tourism plays an important and positive role in international economic and cultural cooperation, and the tourism cooperation relationship between China and North Korea has lasted for more than 30 years. China has become the country with the largest number of tourists to North Korea. However, because the relevant data of tourism to North Korea are not public, it also brings difficulties to the further study. This paper based on the Baidu Index of 31 provinces and regions in China and discusses the temporal and spatial distribution characteristics and influencing factors of travel demands to North Korea. The findings from the research are as follows. First, the travel demands from 2011 to 2018 showed an overall trend of initial increase followed by later decrease. The seasonal difference is significant. The peak season is longer than the off-season. Secondly, on the whole, the travel demands to North Korea showed a spatial agglomeration effect, and the provinces with high demands or low demands gather significantly in space. Taking “Hu line” as the boundary, the east is higher than the west. The hot spot areas and cold spot areas gradually transition from east to west. Thirdly, holidays, population, GDP, per capita disposable income, Internet penetration and education are the main influencing factors of tourism demand to North Korea. By using Baidu Index, this paper overcomes the bottleneck of inaccessible tourism data to North Korea. At the same time, from the perspective of tourist source countries, this paper discusses the spatial-temporal differentiation and influencing factors of travel demands in terms of geographical space, and compares it with existing studies, expanding the research framework of China’s outbound tourism.

## Introduction

Tourism has become the most easily realized and promoted field in regional cooperation due to the attributes of economy and culture [1]. Outbound tourism is an important part of tourism development. With the process of transnational tourism cooperation, border tourism has become a popular choice for tourists to travel abroad because of the convenience and low cost. And thus border tourism is not only conducive to the development of regional integration, but also of great significance to regional cooperation and globalization [2].

North Korea’s tourism cooperation is the only economic industry that is not sanctioned by the United Nations, and the tourism cooperation relationship between China and North Korea has lasted for more than 30 years. The first group of Chinese residents set off from Dandong to make a one-day trip to North Korea in the year 1988 and this preluded the travel to North Korea in China [3]. In 2008, North Korea was officially designated as the destination of organized outbound tours for Chinese residents.

In this situation, the North Korean government attaches great importance to the construction of domestic tourism and cooperation with neighboring countries. After Kim Jong Un came to power, he vigorously developed the economy and attached importance to tourism. In 2016, North Korea’s issued a five-year economic development plan which aims to build it into one of the most powerful countries. In this plan, tourism is a very important area of economic development. North Korean government successively delimits Wencheng Island Tourism Development Zone (2013), Qingshui Tourism Development Zone (2014), Wonshan-Jingangshan International Tourism Zone (2014), Maofeng International Tourism Special Zone (2015), Wonshan Gema Coast Tourism Zone (2018) and other 4 major tourist attractions with their characteristics [4].

Additionally, with the development of border tourism, cooperative relations with China have been continuously deepened. In 2011, the self-driving tour from Yanbian, Jilin, China to Rason, North Korea started [5]. In 2016, a half-day landed tour from Dandong, Liaoning to Xinyizhou was opened [6]. In 2018, because of departure procedures have been simplified, the tourists can travel from Hunchun, Jilin to Rason in half day [7]. Currently, the not-stop flights from Shenyang [8], Beijing [9], Shanghai [10], Xian [11], Chengdu [12] and other cities of China to Pyongyang have been launched. In 2018, with the relaxation of the tense relationship between North Korea and South Korea, the former turned into the main tourist destination for Northeast Asian countries. China ranked number one in North Korea’s main Northeast Asian tourism market to North Korea. Tourism over the last 30 years has deepened the close bilateral relationship between China and North Korea. Travel routes to North Korea were constantly added and the tour coverage expanded. The Chinese tourist market demand for North Korea grew exponentially.

Scholars’ researches on tourism between China and North Korea mainly focuses on the development process, border cooperation and the behavioral characteristics of Chinese tourists to North Korea. Firstly, Kim (2018) viewed the change from 1980 to 2017 between these two countries chronologically. He proposed that the size of China’s tourism to North Korea showed fluctuated. The fluctuation proves that China’s tourism to North Korea is vulnerable to changes in external circumstances. Also, it provides a window through which the relationship between North Korea and China can be observed [13]. Xu & Wen (2020) combed the status quo of North Korea’s tourism development in terms of the construction of special tourism zones, tourism routes, and tourist products, and also discussed the prospects of the tourism zones [2]. Secondly, Wen (2002) discussed the economic and indirect benefits brought by tourism cooperation between China, North Korea and South Korea to the development of the Tumen River region from the perspective of regional cooperation [14]. Man (2010) combed the development process of Dandong, Liaoning with North Korea, analyzed the existing problems, and proposed border cooperation to promote the transformation and upgrading of Liaoning’s border tourism [15]. Chung & Cui (2020) examined the border tourism between China and North Korea. They provoked that border tourists from China to North Korea had become significant economic engines for the development of North Korea’s economy, and the border tourism in the region has been changing from in the form of cross-border tourism to trans-border tourism cooperation [16]. Thirdly, Dong & Jin (2020) conducted 349 questionnaires to analyze the behavioral decisions of Chinese tourists visiting North Korea. The result showed that attitude, subjective norm, positive anticipated emotion, negative anticipated emotion, and the frequency of past behavior have significant influence on desire [17]. Li & Wang (2020) adopted the social contact theory to examine their attitude changes through tourism among 34 Chinese visitors to North Korea. They identified both positive and negative post-trip attitude changes. They provoked that North Korea were considered as a "friendly" neighbour with conflicts [18].

From the literature review, there are more qualitative researches and less quantitative researches. Most of the quantitative researches are conducted with questionnaires, which is not universal. The quantitative research across the whole China is still a research blank, and the non disclosure of North Korea’s data brings difficulties to further study. From the literature review, there is a gap in tourism demands from the perspective of the original tourism country. Moreover, there are more qualitative researches and less quantitative researches. Quantitative analysis can reflect the trend of tourist flows to North Korea clearly, especially in horizontal comparison and changes in different years. To overcome the bottleneck of data acquisition, this article intends to study the travel demands to North Korea based on the network attention—Baidu Index. Previously, many studies have shown that network attention has a close relationship with travel demand. Ma, Sun & Huang, et al. (2011) compared the related data of network attention with urban tourism flow and found that they were correlated, and thus constructed a model to show the relationship between tourism flow and network attention [19]. Huang, Zhang & Ding (2017) discussed the relationship between Internet search data and actual tourist flow. His research shows that Baidu index is positively correlated with tourist flow [20]. Liu, Zhu, Zhag, et al. (2021) proposed a tourism flow forecasting model based on internet attention and verified the effectiveness of the model. He discussed the fluctuation difference and correlation between scenic spot passenger flow and online information flow on the inter day time scale [21]. It can be seen from this that the Baidu Index represents interest on tourist destinations of netizens in the tourist source area. And to a certain extent, it can predict the tourist flow and its trend.

## 2. Materials and methods

### 2.1 Data sources

#### 2.1.1 Baidu index data

The Baidu search engine is the most widely used online search tool among Chinese netizens. The Baidu Index records the search traces of netizens, uses their search keywords to make statistics, and analyzes and calculates the frequency and weighted sum of each search keyword in the search engine. This paper studies the demands to North Korea with Baidu index. Although there are differences between the Baidu index and actual travel demands, it can reflect the basic demands and trends. Related studies have used the Baidu index in the analysis of tourism demands. Ma & Long (2017) use the scenic spots in Hunan Province as keywords, and then use the Baidu Index to measure the tourism demand of the residents in Hunan Province for the scenic spots [22]. Ruan & Li (2018) used "Jiuzhaigou Travel Guide", "Jiuzhaigou Tickets", "Jiuzhaigou Weather", and "Jiuzhaigou Hotels" as keywords to obtain the Baidu Index to measure tourism demand [23]. In addition, Ruan et al. (2019) used "Thailand Travel Guide", "Thailand Travel Notes", "Thailand Tourist Visa", "Bangkok Travel Guide", "Phuket Weather", and "Chiang Mai Travel Guide" as search keywords to discuss the time and space differences of China’s outbound travel demands to Thailand [24].

Based on the researches above, this paper is based on the Baidu index data provided on the platform(https://index.baidu.com/), selecting six keywords that meet the standard of availability, representativeness, and highly attentioned. "North Korea Travel", "North Korea Travel Price", "North Korea Travel Notes", "Pyongyang Weather", "North Korea Currency Exchange Rate", "North Korea Tourism Visa" (keywords with similar meanings, select the one with the most attention) six keywords are searched monthly during 1^st^ January 2011 to 31^st^ December 2018. The six keywords are selected for three reasons: Firstly, since fewer keywords relevant to North Korea have been entered, it is necessary to select keywords that are related to tourism and also with a higher degree of attention from the existing keywords. Secondly, the six words represent the preparation before the tourists travel to North Korea. "Travel to North Korea" reflects the netizens’ concern about traveling to North Korea. "North Korea Travel Notes" includes many aspects such as food, housing, travel, shopping, and entertainment, as well as precautions for traveling to North Korea. "North Korea travel price", "North Korea tourism visa", "North Korea currency exchange rate" reflect the preparation and decision-making behavior before the visit. Pyongyang is the destination with the largest number of Chinese tourists, and it can represent the travel needs of North Korea to the greatest extent. "Pyongyang weather" helps tourists arrange their itineraries better. Thirdly, the Baidu Index platform began to include keywords in 2011, thus 2011–2018 is selected as the research period.

#### 2.1.2 Climate data

In order to analyze the characteristics of time changes, temperature and humidity data are selected. The climate data is sourced from National Oceanic and Atmospheric Administration (NOAA) and National Centers for Environmental Information (NCEI). The name of data set is "Wheat A wheat germ-agri-meteorological big data system V1.4.6". The temporal resolution is monthly average value, and measured by Meteorological station data.

#### 2.1.3 Socio-economic data

In the analysis of influencing factors, the population, per capita disposable income, and education data are obtained from the "China Statistical Yearbook" (http://www.stats.gov.cn/tjsj/ndsj/). The Internet penetration rate is obtained from the "China Internet Development Report" (http://www.cnnic.net.cn/). The related traffic data is collected from the news published on the internet. (Due to different statistical methods, Hong Kong, Macao, and Taiwan are not discussed here).

### 2.2 Research methods

#### 2.2.1 Coefficient of variation

The coefficient of variation is used to measure the degree of variation of the group of data. It is the ratio of the standard deviation of a group of data to its mean and was used to calculate the monthly difference in online attention degree of travel to North Korea. The calculation formula is as follows:

$$C_{V}=\frac{1}{\bar{y}}\sqrt{\sum_{i=1}^{n}(y_{i}-\bar{y}{)}^{2/n}}$$ (1)

Where C_v_ is the coefficient of variation, y_i_ is the attention in the i^th^ month, and $\bar{\text{y}}$ is the mean of y_i_. The larger the C_v_, the greater the monthly difference degree of the online attention degree to travel to North Korea.

#### 2.2.2 Spatial autocorrelation

*Global autocorrelation*. Global spatial autocorrelation is mainly used to determine whether a variable is spatially related. Global spatial autocorrelation can reflect the spatial clustering distribution features of the variable. In the current study, global autocorrelation was taken as an important tool to analyze the spatial representation of Chinese travel demands to North Korea. The objective was to study the spatial correlation and clustering features of the travel demands to North Korea. Moran’s I index is a common test index, and the calculation formula is:

$$I=\frac{\text{n}}{s_{o}}\times \frac{\sum_{i=i}^{n}\sum_{j=1}^{n}w_{ij}(x_{i}-\bar{x})(x_{j}-\bar{x})}{\sum_{i=1}^{n}{\left(x_{i}-\bar{x}\right)}^{2}}$$ (2)

Where I is the Moran’s I index, n is the number of study units, x_i_ and x_j_ represent the attribute values in the i^th^ and j^th^ study units, $\bar{x}$ is the mean of x_i_, s_o_ is the sum of all elements of the spatial weight matrix, and w_ij_ represents the spatial weight matrix of the study units i and j. Moran’s I domain is [−1, 1], in which a value greater than 0 means spatial clustering and a value smaller than 0 means spatial dispersion. When Z(I)>1.96 and P(I)<0.05, there is a significant correlation.

*Local autocorrelation*. Local spatial autocorrelation can depict the spatial differences of the travel demands to North Korea in more details. In the present study, the Getis-Ord G*i index was used to measure the local features of the travel demands to North Korea. The objective was to identify the spatial patterns of cold spot areas and hot spot areas. The calculation formula is as follows:

$$\begin{array}{l} G_{I}^{*}(d)=\sum_{j=1}^{n}w_{ij}x_{j}/\sum_{j=1}^{n}x_{j} \end{array}$$ (3)

Where n is the number of study units, x_i_ and x_j_ are attribute values of space units i and j, and w_ij_ is the spatial weight matrix. A positive and significant G*i suggests a high-level clustering area and the hot spot area of the travel demands to North Korea. If G*i is negative and significant, it belongs to a low-level clustering area and the cold spot area of the travel demands to North Korea.

#### 2.3.3 Panel data model

Combined with the characteristics of cross-section, time, and three-dimensional information of the influencing factors of travel demands, select the panel data model with the help of software stata 16.0 to analyze the spatial influencing factors of travel demands to North Korea (used in 3.2.2), and use F test and Hausman test to determine the setting mode of the panel data model. The calculation formula is as follows:

$$\begin{array}{l} {\text{y}}_{\text{it}}=\alpha_{\text{i}}+\beta_{1i}X_{1it}+\beta_{2}{}_{i}X_{2}{}_{it}+\ldots \beta_{ki}X_{kit}+\mu_{it}\\ \left(i=1,2,3\ldots ,\;\;\;\;N;t=1,2,3\ldots ,\;\;\;T\right) \end{array}$$ (4)

In formula (4), y_it_ is the dependent variable; α_i_ represents the intercepted item of the panel data model; X_1it_…X_kit_t is the independent variable, N is the number of individual members of the cross-section; T is the number of sample observation periods for each section member; β_1i_…β_ki_ represents the coefficient of the explanatory variable.

## 3. Results

### 3.1 Temporal differences

#### 3.1.1 Annual differences

**Fig 1:**
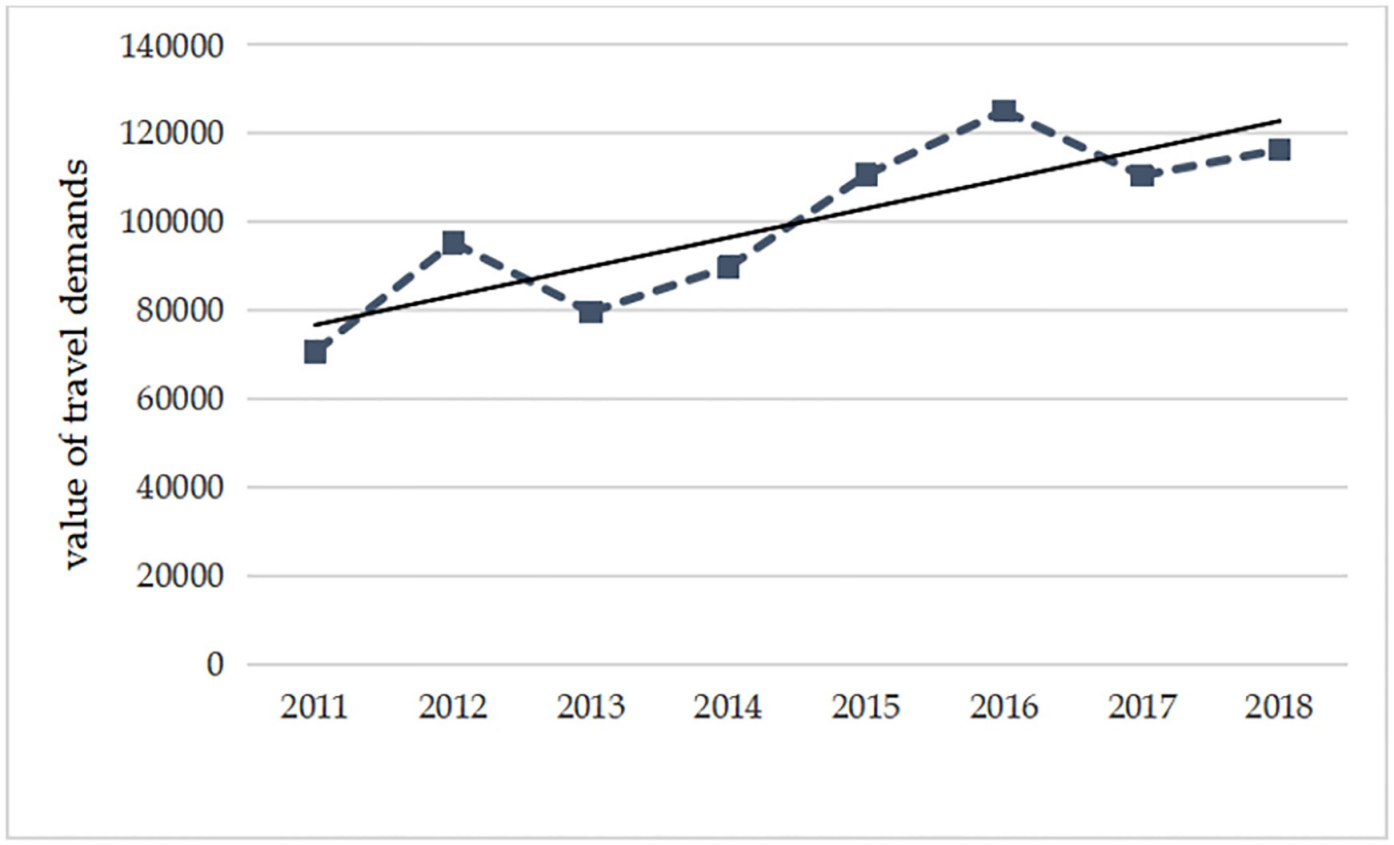
The number of travel demands to North Korea during 2011–2018.

An increase in the travel demands to North Korea was noted from 2011 to 2018 (Fig 1). The travel demands to North Korea showed the fastest increase in 2012. The travel demand increased by 35.02% compared to 2011. In 2013 and 2014, the travel demands to North Korea decreased to some extent, but from 2015, the demands were rapidly on the rise. From 2011 to 2018, travel demands to North Korea in 2016 are much more than in other years. The changes in the travel demands were affected by the international situation and the development and opening-up policies of North Korea. In 2015, North Korea had set a goal of developing and expanding its economy vigorously. A five-year plan for its economic development was formally proposed in 2016, to build North Korea into "one of the five most powerful nations". Tourism was considered an important area of economic development in the development plan, which resulted in nine distinctive tourist areas being set up on its land [25]. In 2018, the leaders of North Korea and South Korea had a successful meeting. This resulted in promoting peace and stability of the Korean Peninsula. This further brought North Korea on the map with a positive image which attracted interest from the international community. The Korean Peninsulas had witnessed a turbulent situation in the past. Tourists were mainly concerned about their safety when sightseeing activities. The situation has gradually improved and it seems to be more stable for tourists. The latter now have fewer safety concerns. This has resulted in more tourist growth; the country has been attracting more international tourists. Tourists became more curious about this magic country and are willing to travel to North Korea.

Tourism has now become an important pillar of the economic development of North Korea. In addition, with the deepening of its opening-up, North Korea has closer cooperation with China in terms of exit and entry. Procedures for traveling to North Korea were simplified and more convenient for Chinese tourists. A pilot program was launched in the year 2010 in Yanbian, located in the Jilin Province in China. Tourists who want to travel to North Korea can get their travel permits at Yanbian public security bureau simply by showing their ID cards and household registration books. In 2016, a program named "landing tour" was launched for Chinese tourists to travel to North Korea from Dandong, Liaoning Province, China. Tourists can now travel to North Korea for half a day without a passport. In 2018, the procedures for travel to North Korea were further simplified at Quanhe Port of Hunchun in the Jilin Province of China. With the submission of relevant application materials and their ID cards, tourists can get their travel permits within one hour. Tourists from Dandong of Liaoning Province and Yanbian of Jilin Province, China, etc. can also travel to North Korea for one, two, or three days only with travel permits, and without the need of a passport. The entry and exit management of travel to North Korea has become more and more beneficial for North Korea and China. The infrastructure supporting the tourism industry is being improved as the tourist regions and travel routes for foreign tourists have increased in North Korea. The author notes that the development and opening-up policies of North Korea have an impact on the travel demands to North Korea in China.

#### 3.1.2 Seasonal and monthly characteristics

**Fig 2:**
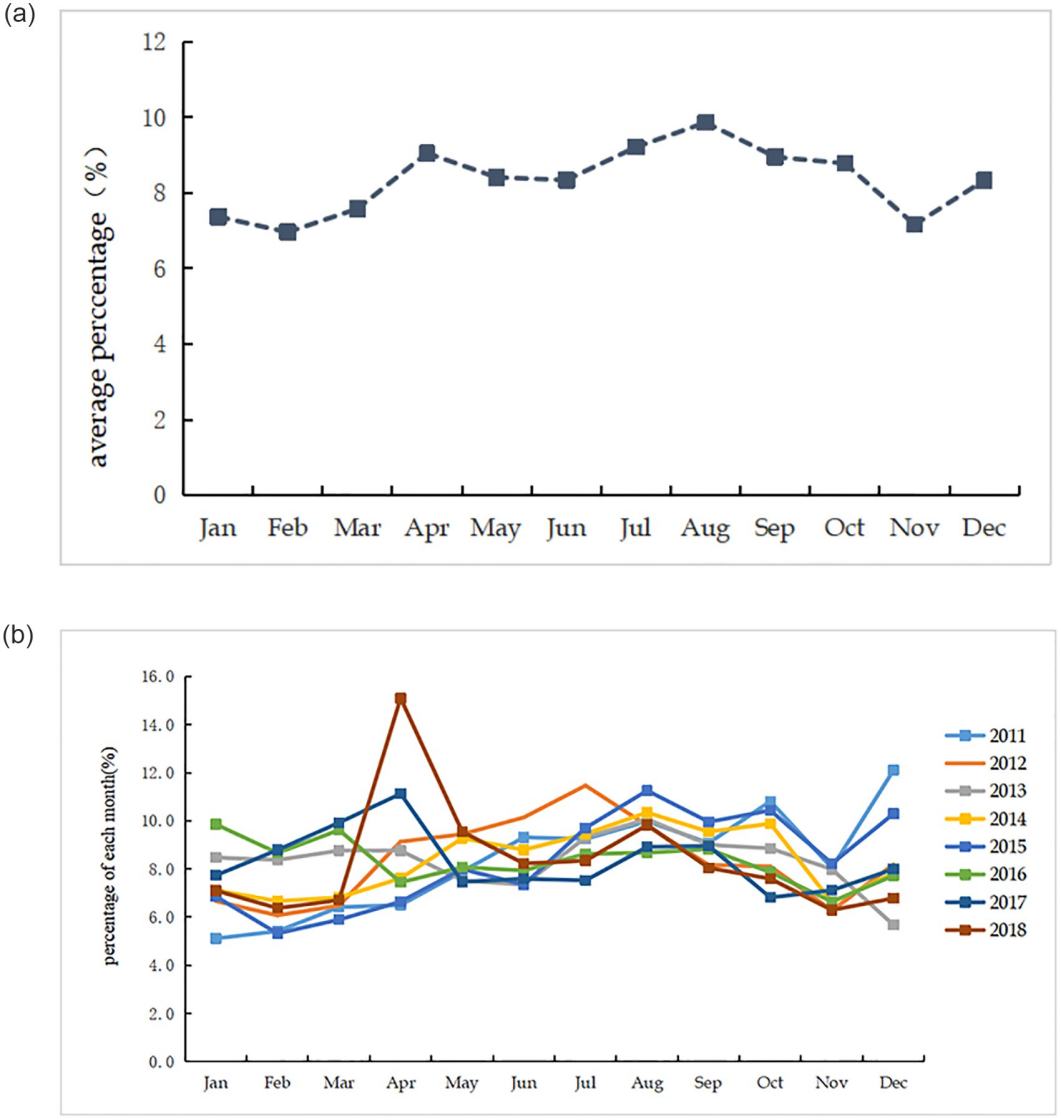
a: The average percentage of each month during 2011-2018. b: The percentage of each month from 2011 to 2018.

Calculate the percentage of each month in the whole year (Fig 2a) and calculate the average percentage of each month in 2011–2018 (Fig 2b), the trend chart of the average monthly demands from 2011 to 2018 is obtained, as shown in Fig 2. Referred to the tourist flow season division standard [26], the months in which the average monthly demands are more than the annual average monthly demand are classified as peak seasons, and the months where the average monthly demand 80%-100% is considered the off-season. According to calculations, it is found that the peak season is from April to October, and the other months are the off-season. The peak season is longer than the off-season, and the seasonal characteristics are significant.

It can be seen from Fig 2(a), that from 2011 to 2018, the travel demands to North Korea increased fast in April, August, and October. From the average percentage of each month shown in Fig 2(b), August was the main peak, and April and October were the second peaks. The travel demands to North Korea increased significantly in these three months. On the contrary, February, June, and November are the three valley periods of the year. Compared with the previous and subsequent months, the demand for tourism to North Korea is significantly lower. There is a great difference between peak and valley periods. By calculating the coefficient of variation of each month from 2011 to 2018, the coefficient of variation of each month from 2011 to 2013 decreased from 0.25 to 0.13, and the difference between each month gradually decreased. The coefficient of variation was 0.16 in 2014 and increased to 0.23 in 2015, with an increase in the difference among months. In 2016, it decreased to 0.10. Combining the coefficient of variation of 2016 and Fig 2(a), travel demands in January, February, and March increased significantly. The difference of each month also decreased significantly. In December 2015, the construction of the landing park area in Sinuiju was completed, and 300 Chinese and North Korean tourists attended the opening ceremony. In January 2016, North Korea officially launched a helicopter tour of Pyongyang. The launch of a series of new projects led to the first wave of growth in travel demands to North Korea in 2016. After 2016, it increased to 0.14 in 2017 and increased to 0.28 in 2018. During 2011–2018, the variation coefficient increased and decreased from time to time, indicating that the inter-annual fluctuation of travel demands between months is very significant. It will be of great significance to the further development of China’s tourism to North Korea in the future by reducing the difference of each month in a year and narrowing the change of each year.

### 3.2 Spatial characteristics

#### 3.2.1 Global spatial pattern

The calculation results of Moran’s I in Formula (3) (Table 1) showed that from 2011 to 2018, Moran’s I was greater than 0, Z (I) index was greater than 1.942, and P (I) index was less than 0.05. An exception is in the year 2016, when the P (I) index was 0.052, indicating that the travel demands to North Korea are not significantly correlated in space. This shows a spatial characteristic of clustering distribution. During the period from 2011 to 2018, the provinces with high demands showed spatial clustering, and the provinces with low demands also showed clustering distribution. This clearly indicates that the travel demands to North Korea from the 31 provinces (cities and autonomous regions) in China were not balanced.

**Table 1:** Characteristics of spatial clustering of travel demands to North Korea.

Year	Moran’s I	Z (I)	P (I)	Spatial pattern
2011	0.090	2.014	0.044	Clustering distribution
2012	0.109	2.048	0.041	Clustering distribution
2013	0.169	2.817	0.005	Clustering distribution
2014	0.190	3.073	0.002	Clustering distribution
2015	0.140	2.405	0.016	Clustering distribution
2016	0.108	1.942	0.052	Random distribution
2017	0.115	2.105	0.035	Clustering distribution
2018	0.215	3.188	0.001	Clustering distribution

From 2011 to 2014, Moran’s I gradually increased from 0.090 to 0.190, suggesting that the imbalance of the travel demands to North Korea from China kept growing. The travel routes were barely known by the tourists in the initial period of development of travel from China to North Korea. North Korea refused all foreign tourists who wanted to travel to North Korea in 2014, to guard against the Ebola virus. Therefore, the development of travel to North Korea was not stable as it was often interrupted due to multiple factors. As a result, only the regions and provinces surrounding the ports of North Korea knew about and showed interest in traveling to North Korea, while the demands from other places remained low. Since 2015, the Moran’s I dropped to 0.140. This implies that the imbalance of the travel demands is reduced. The travel routes from Dandong of Liaoning Province of China to Pyongyang and from Yanbian of Jilin Province of China to Samjiyon and Rason were opened. The travel routes and accommodation capacity for tourists were better than in the preceding years. Travel routes were promoted resulting in an increase in travel demand to North Korea from across the whole country and hence the travel demand became relatively balanced. In 2018, the government leaders of North Korea and South Korea had three meetings, leaders of North Korea and America had their first meeting, and North Korea announced to cease its nuclear test and transcontinental ballistic missile launch. These had positive impacts which made the situation of the Korean Peninsula increasingly stable. In addition, North Korea showed its determination to develop the economy vigorously. Tourism planning and development featured prominently with nine tourist areas in its land, including the Pyongyang area, Mount Kumgang area, Mount Myohyang area, Kaesong area, Nampo area, etc. Travel demands to North Korea hence experienced an increase in 2018. A few of the other provinces with high economic levels also exhibited significantly increased travel demands except for the areas surrounding the ports to North Korea. The travel demands to North Korea were concentrated, and the value of Moran’s I increased.

#### 3.2.2 Local spatial pattern

**Fig 3:**
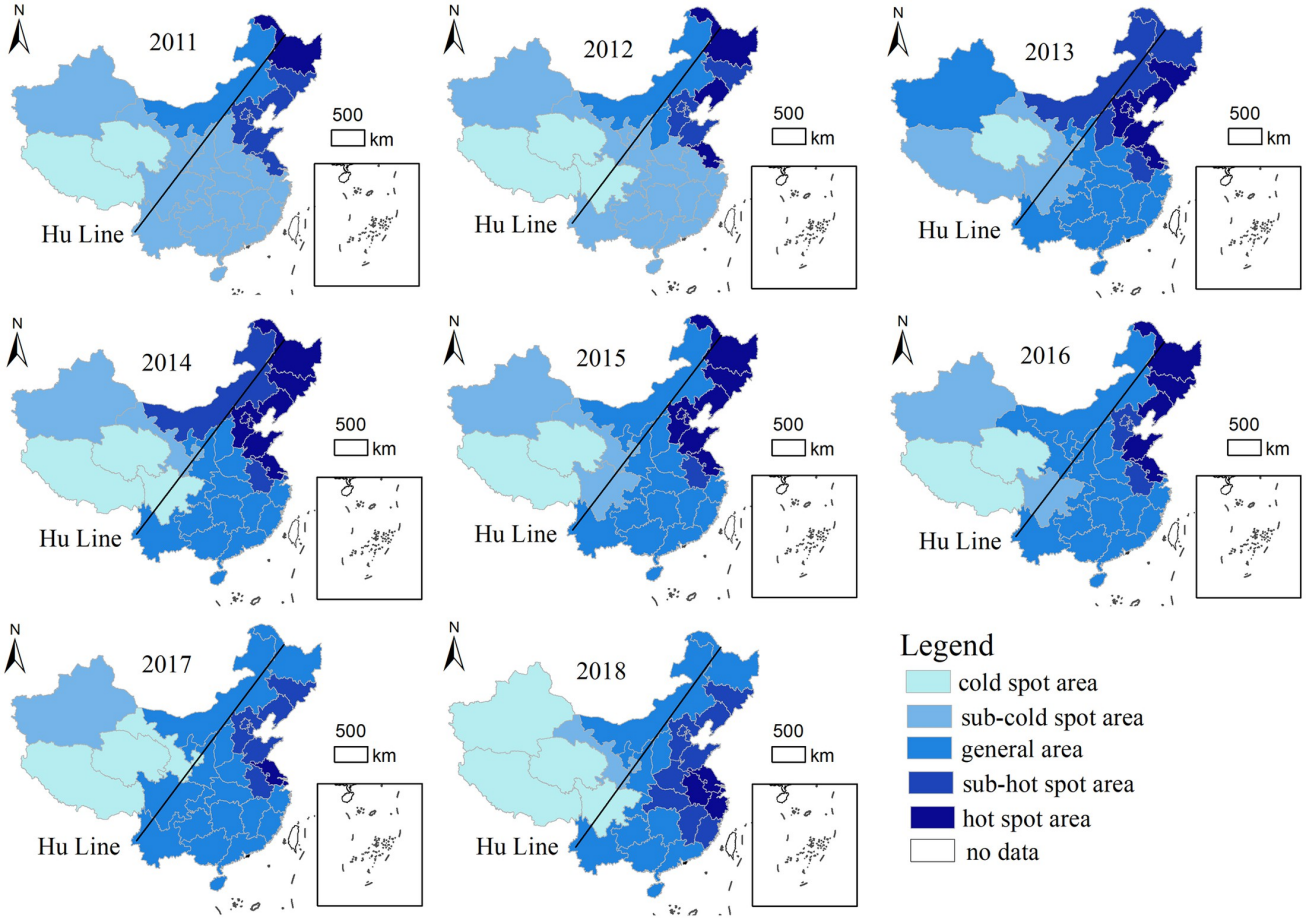
Distribution map of travel demands to North Korea (The administrative boundaries were obtained from the Chinese National Geographic Information Center (http://ngcc.sbsm.gov.cn), using Acrgis 10.6.1 for visual processing. The figure is similar but not identical to the original image and for illustrative purposes only).

The cold and hot spot areas of travel demands to North Korea in 31 provinces (cities and autonomous regions) were analyzed by the hot spot analysis tool, Arcgis 10.6.1 obtain the Getis-Ord G i* index and divide it into 5 categories by Jenks natural segment point, thus generating the cold and hot spot pattern of travel demands to North Korea. It is shown in Fig 3. On the whole, travel demands to North Korea show obvious spatial distribution characteristics of the "Hu line". The line runs from Aihui County, Heihe City, Heilongjiang Province to Tengchong County, Tengchong City, Yunnan Province. Hu Huanyong first named the line in his paper "China’s Population Distribution" published in "Scientia Geographica Sinica", 1935. This line was originally used to describe China’s population distribution. The population density in the southeast of the line is significantly higher than that in the northwest [27,28], and now this line is not only the boundary line of population, but also the boundary line of climate, ecological circumstance, economy, and culture. This line is recognized and quoted by demographers and geographers at home and abroad. (It’s called "Hu Huanyong line" by Professor Tian Xinyuan of Ohio State University).

From 2011 to 2018, the "Heihe-Tengchong Line" ("Hu Line") is taken as a dividing line for travel demands to North Korea. During this period, hot spot and sub-hot spot are mainly distributed in the northeast and eastern coastal areas. The cold and sub-cold spots are mainly concentrated in the western and northwestern regions. Among them, the hot spot and sub-hot spot areas show a trend of gradually expanding, and the cold spot and sub-cold spot area are relatively stable. Firstly, from 2011 to 2013, hot and sub-hot areas grow rapidly. In addition to Heilongjiang Province, Liaoning, Jilin, Hebei, Beijing, Tianjin, Shandong, and Jiangsu provinces have become hot spots, and Inner Mongolia, Shanxi, and Anhui have become sub-hot spots. Secondly from 2014 to 2016, the cold and hot spots alternate, the sub-cold spots and general areas increase, and the hot spots decrease. Xinjiang has changed from a general area to a sub-cold spot area, and Tibet has changed from a sub-cold spot area to a cold spot area. Inner Mongolia has changed from a sub-hot spot to a general area. Beijing, Tianjin, and Hebei have changed from hot spot to sub-hot spot areas. Thirdly, from 2017 to 2018, there are frequent alternations between different regions, the concentration of cold spots and hot spots is more obvious, and the hot spot area migrate to the north. During this period, Sichuan was newly added as a cold spot area, Heilongjiang became a general area, and Liaoning, Jilin, and Shandong become a sub-cold spot area. Shanghai, Zhejiang, and Anhui have gradually become hot spots.

Overall, China’s travel demands to North Korea shows obvious spatial differentiation characteristics. From 2011 to 2018, with the Hu Huanyong Line as the boundary, the hot and sub-hot areas are concentrated in the east, the cold and sub-cold areas are concentrated in the west, and from the east to the west, there is a transition from hot to cold areas. In addition, with the continuous development of tourist destinations in North Korea, there is a new trend of tourism to North Korea in southern regions.

### 3.3 Analysis on the influencing factors

#### 3.3.1 Influencing factors of temporal characteristics

Climate has an impact on tourists’ activities, it may influence by the temperature, relative humidity and sunshine duration [29]. Climatic comfort degree refers to the climatic conditions that people can feel comfortable in the body without resorting to any measures to escape the heat and cold. It is an important environmental factor that affects tourism activities [30] and a natural factor that affects residents’ travel needs [31]. The most commonly used evaluation index is the temperature and humidity index [32].


THI=(1.8t+32)−0.55(1−f)(1.8t−26) (5)

In formula (4), t is the temperature in Celsius; f is relative humidity. See Table 2 for the classification standard of temperature and humidity index.

**Table 2:** Grade standard of moisture-temperature index.

THI	Feelings	Level	THI	Feelings	Level
<40	Extremely cold, uncomfortable	e	65–70	Warm, comfortable	B
40–45	Cold, uncomfortable	d	70–75	hot and uncomfortable	C
45–55	Cold, uncomfortable	c	75–80	Stuffy and uncomfortable	D
55–60	Cool, comfortable	b	>80	Extremely Stuffy and uncomfortable	E
60–65	Cool, very comfortable	A			

This article selects the three major tourist destinations in North Korea, Pyongyang, Raxian, and Kaesong, to calculate the temperature and humidity index for the years of the monthly average temperature (°C) and average relative humidity (%).

In order to accurately understand the impact of climate comfort on the travel demand to North Korea, Assign the level of "e, d, c, b, A, B, C, D, E" (Table 2) in the temperature and humidity index to "1, 3, 5, 7, 9, 5, 3, 1" to make it calculable value.

In addition to climate, holidays also have an important impact on tourism activities [33]. Compared with domestic travel, outbound travel takes longer, and holidays provide time for travel. Considering that the demand often precedes the actual travel activities, travel demands first generate tourism activities, and the influence of the holiday on travel demands is advanced. At the same time, considering that social factors such as "holidays", "summer vacation", and "festival activities" are difficult to quantify. Referring to the related research of Ruan [22] and Zhang [26], according to the actual degree of influence and the advancement of its influence on network attention, use the virtual index to measure its influence. April is the inquiry and preparation stage for the May 1st peak, with a value of 0.5, the value of "May Day" and "National Day" as 1, and the summer vacation of August and September as 0.5. September is the preparation stage of October 1st, but the assignment is not repeated here, and the other months are all 0.

This paper uses climate comfort and holiday system as independent variables, and travel demand to North Korea as dependent variables. With the help of Spss21 software, the least square method is used to perform linear regression analysis. The equation is as follows:

$$P=7.379+0.084C_{\text{i}}+1.1411H_{\text{i}}$$

In the formula, R2 = 0.742, adjustedR2 = 0.684, indicating that the model fits the data well. Among them, the Hi regression coefficient of the holiday system passed the significance test, and the significance level was 0.025, that is, for every 1 unit change in the virtual factor, the demand would increase (or decrease) by 1.1411%; while the climate comfort level Ci failed the significance test. The influencing factors are not established.

The current study’s analysis showed that the peak seasons of travel demands to North Korea in China were consistent with Chinese holidays. This implies that the Chinese system has a great impact on Chinese travel demands to North Korea. The residents in Liaoning and Jilin provinces are capable of arriving at and returning from the location of the tourism port to North Korea within one day. Residents of the remaining provinces however do require more travel time. It is noted that a new tourism market is emerging. Tourists have started independent tours, such as self-driving tours and self-guided tours, and hence travel time will be considerably more. This requires that tourists plan for adequate traveling time. Hence, tourists seem to prefer traveling to North Korea over the holidays including on Labor Day, National Day, Mid-Autumn Festival, and during the summer vacation.

As seen from Fig 2(b), the demands to North Korea mainly appear in April, July, August and October, of which July and August are the peaks and April and October are the secondary peaks. "Labor Day" holiday is the first long holiday after the Spring Festival. People’s travel demands are relatively high, and the travel demands to North Korea discussed in this paper are before tourism activities, so there is a small peak in April. July and August are China’s summer holidays, and a large number of parents take their children out to travel. October 1-7^th^ is the National Day golden week, which provides enough time for people to travel abroad. Even if the Liaoning and Jilin provinces spend less time traveling abroad, tourists from the other 29 provinces and regions still need to spend more time traveling to North Korea. Therefore, more time guarantee has an important impact on the travel demands to North Korea. China’s holiday system can meet the time requirements of outbound tourism, which is the main factor affecting the time evolution of outbound travel demands.

#### 3.3.2 Influencing factors of spatial characteristics

The influencing factors of travel demands include destination, source of tourists, and their relationships. In terms of the destination, considering that there are few cities in North Korea that could visit and they have certain homogeneity. Thus the destination factors and tourist preference factors are not considered here.

Through regression analysis, Zhang (2016) provoked that the economic intensity, per capita GDP, and the distance between the two places are important factors affecting the spatial distribution of network attention [26]. Sun (2018) proposed that population size, Internet development level, regional economic development level, and education level have a significant impact on the attention of tourism security network [34]. Zhou (2021) proposed that distance, education level, population size and Internet penetration have an impact on the network attention of Pingyao ancient city [35]. Ruan Wenqi (2019) using geographic detectors, set up detection factors such as economic development level, population size, international tourism openness, external transportation, external economic intensity, industrial structure, informatization degree and geographical location [22]. Based on researches before, combined with the actual situation of tourism to North Korea and data availability, an index system of influencing factors of travel demands to North Korea is constructed from tourist sources (X1, X2, X3, X4, and X5) and the relationship between source and destination (X6). Considering transportation conditions have an impact on travel demands. Whether there are nonstop flights or railways between cities in China and North Korea makes a difference in the travel convenience. At present, there are only 5 cities in China that have direct traffic (non-stop flights or railways) to North Korea. In order to reflect the role of traffic and calculate in the panel data model, therefore, the statistical method of the numerical value is adopted, and the provinces in the year with direct flights or railways are recorded as 1, and those that have not been opened are recorded as 0. Thus the indexes are shown in Table 3.

**Table 3:** Influencing factors of travel demands to North Korea.

variable	classification	index	name
explanatory variable	Population size	Residents population	X1
	Economic level	GDP (hundred million yuan)	X2
		Per-capita disposable income (yuan)	X3
	Internet development	Internet penetration (%)	X4
	Education	population of college education or above	X5
	Traffic	Direct transportation to North Korea	X6
explained variable	Travel demands to North Korea	Baidu Index	Y

*X6 uses dummy variables. If any, recorded as 1, and if none, recorded as 0.

In this paper, ADF and HT tests are carried out on X1, X2, X3, X4, X5, X6, and Y. The results show that P < 0.05, rejecting the original hypothesis of unit root, so it is a stationary variable. Meanwhile, using cointegration test, P value < 0.05, rejects the original hypothesis, there is a cointegration relationship between variables, which can be studied in the next step.

Since the data values of different indicators vary greatly, logarithm is selected for X1-X5, X6 is a virtual value. In this model, the logarithm processing is not carried out. The form of panel data model in this study is:

$$\begin{array}{l} \text{Y}=\alpha_{\text{i}}+\beta_{1i}X_{1it}+\beta_{2}{}_{i}X_{2}{}_{it}+\ldots \beta_{ki}X_{kit}+\mu_{it}\\ \left(i=1,2,3\ldots ,\;\;\;\;N;t=1,2,3\ldots ,\;\;\;\;T\right) \end{array}$$

In order to determine the specific form of the model, F test and Hausman test are used to judge the panel model form applicable to the data, and F test and Hausman test are used to judge the panel model form applicable to the data. The p value of F test is 0.0000, which rejects the original hypothesis of establishing the mixed effect model. The result of Hausman also rejects the original hypothesis of establishing the random effect model. Therefore, the individual fixed effect model is selected to estimate the panel data.

From the results (Table 4), X1-X5 are significant (P < 0.05), while X6 is not significant (P > 0.05).

**Table 4:** Result of the panel data model.

variable	coefficient	T	P
X1	18266.11	2.37	0.019
X2	5143.56	3.67	0.000
X3	3825.72	3.10	0.002
X4	4628.38	3.25	0.001
X5	786.38	3.67	0.000
X6	2082.11	1.66	0.098
C	-418559.6	-3.21	0.002

Based on the panel data results, the influencing factor model of travel demands to North Korea can be obtained as:

$$\begin{array}{l} Y=\alpha_{\text{i}}+188266.11\text{ln}X_{1}+\text{5143}{\text{.56lnX}}_{2}+3825.72\text{ln}X_{3}\\ +4628.38\text{ln}X_{4}+786.38\text{ln}X_{5}+2082.11X_{6}+\mu \end{array}$$

From the results, the travel demands to North Korea are mainly affected by four aspects: population, economic development, Internet development level, and education level.

Regional population (X1) has a significant positive effect on travel demands to North Korea, and its influence coefficient is the largest (18266.11), that is, the total population of the region changes by 1%, and the demand for tourism to North Korea increases or decreases by 182.66. Since Baidu Index is based on the data generated by the search records of internet users, the number of views is closely related to the population size of the region. The research results also confirm that the larger the population, the greater travel demands to North Korea. In addition, the population of each province is large, so the value of its impact coefficient is also large.

In terms of economic development, both GDP (X2) and Per-capita disposable income (X3) have an impact on tourism demand in North Korea. GDP (X2) has a significant positive effect on the travel demands to North Korea. Its influence coefficient is 5143.56. That is, GDP increases by 1%, and the demands increases by 51.43. GDP is one of the main influencing factors of the travel demands to North Korea. GDP is one of the important indicators to measure the economic development level of a region. The higher the regional GDP is, the higher the economy development level of a region will be. That also indicates infrastructures and Internet facilities will be higher. Therefore, the channels for local residents to obtain information about outbound tourism to North Korea through the Internet will be more and more smooth.

Per-capita disposable income (X3) generally refers to the sum of final consumption expenditure and savings available to residents, that is, the income available to residents for free disposal. Per-capita disposable income is the average value of disposable income of residents in a region. GDP represents the overall economic development level of a region, while per capita-disposable income reflects the consumption capacity of residents in a region for non-necessities. It has a positive effect on travel demands to North Korea, and the coefficient is 3825.72, that is, X3 increases by 1%, and the demands increases by 38.26. According to Maslow’s demand hierarchy theory, travel demands is at a higher level. Only when residents have enough money in addition to living expenses will they have the willingness to travel. Therefore, the higher the per capita-disposable income, the stronger their willingness to travel to North Korea. On the contrary, residents in some regions will be less willing to travel because of their low disposable income and low consumption capacity of daily necessities.

Internet development (X4) also has a positive effect on the travel demands to North Korea, with a coefficient of 4628.38. The internet penetration increases or decreases by 1%, travel demands will increase(decrease) by 46.28. On the one hand, the Baidu index used in this paper is closely related to the Internet. Regions with higher Internet penetration show that it is more convenient for residents to surf the Internet. On the other hand, the development level of the Internet also reflects the economic foundation of the region to a certain extent. Residents have a strong economic foundation to afford tourism activities.

Education level (X5) has a positive impact on the travel demands to North Korea, with an impact coefficient of 786.38, that is, if the educated the population increases 1%, the travel demands to North Korea will increase by 7.86. To some extent, the education level affects the netizens’ use of the Internet and obtaining information from it. Skilled use of the Internet and effective will be helpful for tourists to make decisions. AS for transportation (X6), the impact is not significant. Traffic (X6) has no significant impact. Whether there is direct traffic to North Korea has no significant impact on travel demands to North Korea.

## 4. Conclusion and discussion

The research on Baidu Index and tourism demands mostly focus in domestic tourism demands, while this study uses Baidu Index to conduct outbound tourism demands research. By using Internet big data, Baidu Index, it’s beneficial to overcome the obstacles to academic researches caused by obtaining data of North Korea. From the perspective of the original tourism country, this paper discusses the temporal and spatial distribution of tourism demands in North Korea and analyzes the influencing factors. The main conclusions of this study are: (1) China’s tourism demands to North Korea fluctuated from 2011 to 2018, but the overall trend was on the rise. The peak season of tourism demands is obvious. April to October is the peak season, of which July and August are the most in-demand. Other months are off-season, and the peak season is longer than the off-season. The needs of "holiday travel" and "leisure travel" are obvious. (2) The demands to North Korea has an agglomeration effect in space. Provinces and regions with high demands tend to be close in space, and the provinces with low demands also tend to approach. Two regions are bounded by the Hu Huanyong Line, and the east of the Hu Line is significantly higher than the west. The hot spot areas and cold spot areas gradually transition from east to west. (3) From the perspective of spatial influencing factors, population size, economic level, Internet penetration, and transportation factors have a high degree of influence on the demands to North Korea, while education has a relatively small impact on the travel demands. From the point of time-varying influencing factors, China’s holiday has an important impact on tourism demands during the year, while climate reasons have little impact on tourism demands to North Korea. This finding is different from the found in the other researches.

This study draws on the research results related to the domestic tourism demands, and initially tries to use the Baidu index to study the outbound tourism demands, expands the application scope of the Baidu index in the research of tourism demand, and expands the period of the study. This paper discusses the dynamic changes in tourism demands to North Korea from one dimension and uses Stata software to conduct panel data analysis over the past 8 years, which more accurately analyzes the influencing factors and effect of tourism demands. At the same time, Baidu Index is selected based on the six elements of tourism to improve the effectiveness and rigor of data acquisition. Through the analysis, it can be seen that the tourism demands to North Korea have an obvious low and high season, which is consistent with Coshall & Chalesworth’s [36] research, which has an obvious correlation with China’s vacation system. In addition, scholars have also verified that climate comfort is an important factor affecting the temporal change of domestic tourism demands, but the results of this study are not consistent with this. Except for residents of Jilin and Liaoning provinces, tourists from other provinces take a long time to travel to North Korea, which is limited by time factors. Among the spatial influencing factors, population, economic level, and informatization degree are important factors that influence the demands to outbound tourism, and these three variables also have a significant impact on the demands to North Korea. In order to improve the reliability of the results, this study selects two indicators of GDP and Per-capita disposable income from the perspective of regional economic development and the money that tourists can use for traveling to discuss the impact of the economy. The study also shows that traffic has a significant impact on the demands to North Korea. Due to the particularity of North Korea, the traffic with cities of North Korea can not only for tourists’ convenience but also have a positive effect on tourism promotion.

At present, there are some limitations in this research. Firstly, considering the particularity of North Korea in international cooperation, the development of border tourism between China and North Korea is significantly affected by the international situation and political factors. There are data limitations to quantify these factors. In addition, there are still limitations in the climate data of major tourist cities in North Korea, and panel data cannot be used to analyze time-influencing factors. Thirdly, the tourism demands to North Korea between different provinces in China at different periods need to be further discussed in detail. In the future, based on the problems above, the research on the tourism demands could be further improved.
